# *Boswellia serrata* intoxication manifesting with syndrome of inappropriate antidiuretic hormone secretion, hyponatremia, seizure, and rhabdomyolysis

**DOI:** 10.62675/2965-2774.20240049-en

**Published:** 2024-05-27

**Authors:** Josef Finsterer

**Affiliations:** 1 Neurology and Neurophysiology Center Vienna Austria Neurology and Neurophysiology Center - Vienna, Austria.

**Keywords:** Boswellia, Drug-related side effects and adverse reactions, Seizures, Rhabdomyolysis, Hyponatremia, Inappropriate ADH syndrome

## Abstract

*Boswellia serrata* is an herbal extract from the *Boswellia serrata* tree that has anti-inflammatory and analgesic properties and alleviates pain caused by rheumatoid arthritis, gout, osteoarthritis, and sciatica. Syndrome of inappropriate antidiuretic hormone secretion accompanied by hyponatremia, seizures, and rhabdomyolysis as a manifestation of *Boswellia serrata* intoxication has not been reported previously. A 38-year-old female suffered clinically isolated syndrome and has since been regularly taking *B. serrata* capsules (200mg/d) to strengthen her immune system. She experienced hypersensitivity to light, ocular pain, nausea, dizziness, and lower limb weakness four days after receiving her first BNT162b2 vaccine dose, and she increased the dosage of *B. serrata* to five capsules (1000mg/d) one week after vaccination. After taking *B. serrata* at a dosage of 1000mg/d for 3 weeks, she was admitted to the intensive care unit because of a first, unprovoked generalized tonic–clonic seizure. The patient's workup revealed syndrome of inappropriate antidiuretic hormone secretion, which resolved completely upon treatment and discontinuation of *B. serrata.* In summary, *B. serrata* potentially causes syndrome of inappropriate antidiuretic hormone secretion when it is taken at high doses. Patients should not self-medicate.

## INTRODUCTION

*Boswellia serrata* is an herbal extract from the Boswellia serrata tree.^(1)^ It has anti-inflammatory and analgesic properties and alleviates pain associated with rheumatoid arthritis, gout, osteoarthritis, and sciatica.^(2)^ There is also evidence that some components of the extract have antiseizure and antiasthmatic effects or may be beneficial for treating collagenous colitis.^(2)^ *B. serrata* is generally well tolerated and has few side effects. Although in vitro and animal studies have been performed,^(3)^ the clinical effects of the extract in humans are uncertain. To date, there is no evidence that *B. serrata* can cause syndrome of inappropriate antidiuretic hormone secretion (SIADH) accompanied by hyponatremia, seizures, and rhabdomyolysis.

## CASE REPORT

The patient is a 38-year-old female with a history of left-sided optic neuritis diagnosed at age 30, leading to the diagnosis of clinically isolated syndrome; complete recovery of visual impairment was achieved with steroid treatment. Since then, she has been regularly taking *B. serrata* capsules (200mg/d, manufacturer-recommended dosage) to strengthen her immune system. She experienced hypersensitivity to light, ocular pain, nausea, dizziness, and lower limb weakness four days after receiving her first BNT162b2 vaccine dose in July 2021, and she increased the dosage of *B. serrata* to five capsules (1000mg/d) one week after vaccination. After taking *B. serrata* at a dosage of 1000mg/d for 3 weeks, she was admitted to the intensive care unit (ICU) because of a first, unprovoked generalized tonic–clonic seizure. An examination revealed serum hyponatremia (112mmol/L (n, 135 - 150mmol/L)), a urine sodium concentration of 58mmol/L, a serum osmolality of 234mosm/kg (n, 280 - 300mosm/kg), a urine osmolality of 739mosm/kg (n, 450- 600mosm/kg), an ACTH concentration of 85,9pg/mL (n, 7.2 - 63.3pg/mL), a normal basal cortisol concentration, a normal C-reactive protein (CRP) concentration, a leucocyte count of 11.4 (n, < 10/l), neutrophilia, lymphopenia, and rhabdomyolysis (a maximum creatine kinase (CK) concentration of 76348U/L (n, 1 - 145U/L)). Cerebral magnetic resonance imaging (MRI) revealed three periventricular, nonenhanced lesions that were unchanged in number and extent compared to those on the previous MRI four years prior. The pituitary gland was normal. Screening for malignancy was noninformative. The patient was diagnosed with SIADH and treated with levetiracetam, forced diuresis, and sodium chloride infusions. After three weeks of treatment and discontinuation of the *B. serrata* capsules, she fully recovered.

## DISCUSSION

This case is interesting because the patient developed SIADH, which manifested as hypoosmolar hyponatremia, most likely triggered by an overdose of *B. serrata*. Alternative causes of SIADH, such as hypothalamic or pituitary lesions, hypothyroidism, hypocorticism, heart failure, or malignancy, were excluded in this patient.

Whether SIADH in this patient was caused by severe acute respiratory syndrome coronavirus 2 (SARS-CoV-2) vaccination remains unclear. Only a few cases of SARS-CoV-2 vaccination-related SIADH have been reported. However, due to the four-week latency between receiving the vaccination and the seizure and the fact that SIADH is an extremely rare complication of SARS-CoV-2 vaccination, a causal relationship is unlikely. The cerebral lesions visible on MRI were excluded as the cause of SIADH because they did not involve the hypothalamus or pituitary gland. Furthermore, SIADH has rarely been reported in association with multiple sclerosis.^(4)^ Given these findings, it cannot be ruled out that the *B. serrata* extract was responsible for SIADH and its complications. Although *B. serrata* usually does not cause any major side effects, some patients report stomach pain, nausea, diarrhea, headache, heartburn, and itching. There is also evidence that *B. serrata* has a natriuretic effect.^(5)^ Intoxication may have occurred because the *B. serrata* concentration in the capsules was greater than usual.

## CONCLUSION

In summary, it is possible that *Boswellia serrata* extract causes syndrome of inappropriate antidiuretic hormone secretion when taken in high doses.

## Data Availability

The data that support the findings of the study are available from the corresponding author upon request.
