# Supplementary material for: Boswellia serrata intoxication manifesting with syndrome of inappropriate antidiuretic hormone secretion, hyponatremia, seizure, and rhabdomyolysis
Source: Crit Care Sci. 2024 May 27;36:e20240049en. doi: 10.62675/2965-2774.20240049-en (PMC11152439; doi:10.62675/2965-2774.20240049-en)
Supplement: Translation

# Translation: Intoxicação por *Boswellia serrata* manifestando-se com síndrome da secreção inapropriada de hormônio antidiurético, hiponatremia, convulsão e rabdomiólise

**DOI:** 10.62675/2965-2774.20240049-pt

**Authors:** Josef Finsterer

**Affiliations:** 1 Viena Áustria Neurology and Neurophysiology Center - Viena, Áustria.

**Keywords:** Boswellia, Efeitos colaterais e reações adversas relacionadas a medicamentos, Convulsões, Rabdomiólise, Hiponatremia, Síndrome de secreção inadequada de HAD

## Abstract

*Boswellia serrata* é um extrato herbal da árvore *Boswellia serrata* que possui propriedades anti-inflamatórias e analgésicas e alivia a dor ciática e causada por artrite reumatoide, gota, osteoartrite. Não há relato na literatura de síndrome da secreção inapropriada do hormônio antidiurético, acompanhada por hiponatremia, convulsões e rabdomiólise, como manifestação de intoxicação por *Boswellia serrata*. Uma mulher de 38 anos diagnosticada com síndrome clinicamente isolada tomava regularmente cápsulas de *B. serrata* (200mg/dia) para fortalecer seu sistema imunológico. Ela desenvolveu hipersensibilidade à luz, dor ocular, náusea, tontura e fraqueza nos membros inferiores 4 dias após tomar a primeira dose da vacina BNT162b2 e aumentou a dose de *B. serrata* para 1.000mg/dia 1 semana após a vacinação. Após tomar *B. serrata* na dose de 1.000mg/dia por 3 semanas, ela foi internada na unidade de terapia intensiva devido à convulsão tônico-clônica generalizada não provocada. A investigação diagnóstica revelou síndrome da secreção inapropriada de hormônio antidiurético, que se resolveu completamente após tratamento e interrupção do uso de *B. serrata*. Em resumo, é possível que *B. serrata* cause síndrome da secreção inapropriada do hormônio antidiurético quando tomada em doses elevadas. Os pacientes não devem se automedicar.

## INTRODUÇÃO

*Boswellia serrata* é um extrato herbal da árvore *Boswellia serrata*.^(1)^ Possui propriedades anti-inflamatórias e analgésicas e alivia a dor ciática e causada por artrite reumatoide, gota e osteoartrite.^(2)^ Há evidências de que alguns componentes do extrato também possuem propriedades anticonvulsivantes e antiasmáticas ou podem ser benéficos para o tratamento de colite colagenosa.^(2)^ *B. serrata* costuma ser bem tolerada e apresenta poucos efeitos colaterais. Embora estudos *in vitro* e em animais tenham sido realizados,^(3)^ os efeitos clínicos do extrato em humanos são pouco conhecidos. Até o momento, não há evidências de que *B. serrata* possa causar síndrome da secreção inapropriada de hormônio antidiurético (SIHAD) acompanhada de hiponatremia, convulsões e rabdomiólise.

## RELATO DE CASO

Mulher de 38 anos com história de neurite óptica do lado esquerdo diagnosticada aos 30 anos, com síndrome clinicamente isolada, obteve recuperação completa da deficiência visual com tratamento com esteroides. Desde então, tomava regularmente cápsulas de *B. serrata* (200mg/dia, dose recomendada pelo fabricante) para fortalecer seu sistema imunológico. Ela desenvolveu hipersensibilidade à luz, dor ocular, náusea, tontura e fraqueza nos membros inferiores 4 dias após tomar a primeira dose da vacina BNT162b2 em julho de 2021 e aumentou a dose de *B. serrata* para cinco cápsulas (1.000mg/dia) 1 semana após a vacinação. Após tomar *B. serrata* na dose de 1.000mg/dia por 3 semanas, a paciente sofreu uma convulsão tônico-clônica generalizada não provocada e foi internada na unidade de terapia intensiva. A investigação revelou hiponatremia sérica (112mmol/L [n, 135 - 150mmol/L]), concentração de sódio urinário de 58mmol/L, osmolaridade sérica de 234mosm/kg (n, 280 - 300mosm/kg), osmolaridade urinária de 739mosm/kg (n, 450 - 600mosm/kg), concentração de ACTH de 85,9pg/mL (n, 7,2 - 63,3pg/mL), concentração normal de cortisol basal, concentração normal de proteína C-reativa, contagem de leucócitos de 11,4 (n, < 10/l), neutrofilia, linfopenia e rabdomiólise (concentração máxima de creatina quinase de 76348U/L (n, 1 - 145U/L). Realizou ressonância magnética do crânio (RMC), que revelou três lesões periventriculares, não realçadas, que não mudaram em número e nem em extensão em comparação com a RMC feita 4 anos antes. A glândula pituitária estava normal. A investigação de malignidade foi pouco esclarecedora. A paciente foi diagnosticada com SIHAD e tratada com levetiracetam, diurese forçada e infusões de cloreto de sódio. Após 3 semanas de tratamento e interrupção do uso de cápsulas de *B. serrata*, a paciente se recuperou completamente.

## DISCUSSÃO

Este caso é interessante porque a paciente desenvolveu SIHAD, que se manifestou como hiponatremia hipo-osmolar, muito provavelmente desencadeada por uma overdose de *B. serrata*. Causas alternativas de SIHAD, como lesões hipotalâmicas ou hipofisárias, hipotireoidismo, hipocortisolismo, insuficiência cardíaca e malignidade, foram descartadas.

Não se sabe se, nessa paciente, a SIHAD foi causada pela vacinação contra a infecção pelo coronavírus com síndrome respiratória aguda grave 2 (SARS-CoV-2). Apenas alguns casos de SIHAD relacionada à vacinação contra SARS-CoV-2 foram relatados. No entanto, devido ao intervalo de 4 semanas entre a vacinação e a convulsão e pelo fato de a SIHAD ser uma complicação extremamente rara da vacinação contra SARS-CoV-2, uma relação causal é improvável. As lesões cerebrais identificadas na RMC foram descartadas como causa de SIHAD porque não envolviam o hipotálamo e nem a glândula pituitária. Além disso, raramente tem-se relatado SIHAD em associação com esclerose múltipla.^(4)^ Considerando esses achados, não se pode descartar que o extrato de *B. serrata* tenha sido responsável pela SIHAD e suas complicações. Embora *B. serrata* geralmente não cause efeitos colaterais graves, alguns pacientes relatam dor de estômago, náusea, diarreia, dor de cabeça, azia e coceira. Há também evidências de que *B. serrata* tem efeito natriurético.^(5)^ A intoxicação pode ter ocorrido porque a paciente utilizou uma concentração de *B. serrata* maior do que o usual.

## CONCLUSÃO

É possível que o extrato de *Boswellia serrata* cause síndrome da secreção inapropriada de hormônio antidiurético quando tomado em doses elevadas.

## Data Availability

Os dados que apoiam as conclusões do estudo estão disponíveis mediante solicitação ao autor correspondente.
